# P^2^T^2^: Protein Panoramic annoTation Tool for the interpretation of protein coding genetic variants

**DOI:** 10.1093/jamiaopen/ooab065

**Published:** 2021-08-07

**Authors:** Elias DeVoe, Gavin R Oliver, Roman Zenka, Patrick R Blackburn, Margot A Cousin, Nicole J Boczek, Jean-Pierre A Kocher, Raul Urrutia, Eric W Klee, Michael T Zimmermann

**Affiliations:** Clinical and Translational Sciences Institute, Medical College of Wisconsin, Milwaukee, Wisconsin 53226, USA; Department of Health Science Research, Division of Biomedical Statistics and Informatics, Mayo Clinic, Rochester, Minnesota, USA; Center for Individualized Medicine, Mayo Clinic, Rochester, Minnesota, USA; Department of Health Science Research, Division of Biomedical Statistics and Informatics, Mayo Clinic, Rochester, Minnesota, USA; Clinical and Translational Sciences Institute, Medical College of Wisconsin, Milwaukee, Wisconsin 53226, USA; Center for Individualized Medicine, Mayo Clinic, Jacksonville, Florida, USA; Department of Health Science Research, Division of Biomedical Statistics and Informatics, Mayo Clinic, Rochester, Minnesota, USA; Center for Individualized Medicine, Mayo Clinic, Rochester, Minnesota, USA; Department of Health Science Research, Division of Biomedical Statistics and Informatics, Mayo Clinic, Rochester, Minnesota, USA; Center for Individualized Medicine, Mayo Clinic, Rochester, Minnesota, USA; Department of Health Science Research, Division of Biomedical Statistics and Informatics, Mayo Clinic, Rochester, Minnesota, USA; Center for Individualized Medicine, Mayo Clinic, Rochester, Minnesota, USA; Genomic Sciences and Precision Medicine Center, Medical College of Wisconsin, Milwaukee, Wisconsin, USA; Department of Biochemistry, Medical College of Wisconsin, Milwaukee, Wisconsin 53226, USA; Department of Surgery, Medical College of Wisconsin, Milwaukee, Wisconsin, 53226, USA; Department of Health Science Research, Division of Biomedical Statistics and Informatics, Mayo Clinic, Rochester, Minnesota, USA; Center for Individualized Medicine, Mayo Clinic, Rochester, Minnesota, USA; Clinical and Translational Sciences Institute, Medical College of Wisconsin, Milwaukee, Wisconsin 53226, USA; Genomic Sciences and Precision Medicine Center, Medical College of Wisconsin, Milwaukee, Wisconsin, USA; Department of Biochemistry, Medical College of Wisconsin, Milwaukee, Wisconsin 53226, USA

**Keywords:** high-throughput nucleotide sequencing, genetic variation, protein annotations, molecular sequence annotation, data aggregation

## Abstract

**Motivation:**

Genomic data are prevalent, leading to frequent encounters with uninterpreted variants or mutations with unknown mechanisms of effect. Researchers must manually aggregate data from multiple sources and across related proteins, mentally translating effects between the genome and proteome, to attempt to understand mechanisms.

**Materials and methods:**

P^2^T^2^ presents diverse data and annotation types in a unified protein-centric view, facilitating the interpretation of coding variants and hypothesis generation. Information from primary sequence, domain, motif, and structural levels are presented and also organized into the first Paralog Annotation Analysis across the human proteome.

**Results:**

Our tool assists research efforts to interpret genomic variation by aggregating diverse, relevant, and proteome-wide information into a unified interactive web-based interface. Additionally, we provide a REST API enabling automated data queries, or repurposing data for other studies.

**Conclusion:**

The unified protein-centric interface presented in P^2^T^2^ will help researchers interpret novel variants identified through next-generation sequencing. Code and server link available at github.com/GenomicInterpretation/p2t2.

## BACKGROUND AND SIGNIFICANCE

High-throughput sequencing is increasingly applied in clinical settings to establish precise genomic diagnoses, and in research, either to understand mechanisms of diagnostic, actionable, or pathogenic variants, or for better understanding opportunities for intervention. However, most variants have limited utility in these applications since they lack functional characterization. For lack of characterization, they are classified as variants of uncertain significance (VUS). Though guidelines^1–3^ aid in interpreting these variants, new data and resources regularly emerge that provide additional information. In practice, interpreting variants can thus become a manual data aggregation procedure that is repeated for each case or genetic variant, often using the same resources. Therefore, data science approaches that integrate multiple types of information are critical for comprehensive understanding, particularly for rare variants where co-observation is unlikely. Since most existing tools focus on the genomic change rather than on the effect of the genomic change in the context of the encoded gene product, new tools are critically needed to help interpret VUS and pathogenic variation alike.

Assembling data for each variant is challenging and time-consuming; relevant literature and databases are typically queried manually, which can result in data being overlooked or underutilized. Tools have been developed for this purpose and generally fit into one of three categories (i) protein-level databases of pathogenicity, functional sites^4^ and domains,^5–8^ population allele frequency,^9^ and post-translational modifications.^10^^,^^11^ Understanding the effect of a genomic variant on the translated gene product is easier when data are presented in the protein context, rather than on the genome. (ii) Natural language processing (NLP) based literature mining tools, which can extract disease-gene-variant associations from research indexed by PubMed,^12^ systematically and uniformly identifying, structuring, and searching the entire public publication record. These tools include concept maps for gene aliases and more, providing a more comprehensive, uniform, and systematic solution compared to manual investigation. (iii) Knowledge transfer from related proteins such as human paralogs.^6^ For example, in paralog annotation analysis (PAA),^13^^,^^14^ a multiple sequence alignment (MSA) identifies analogous residues in a family of proteins, enabling information to be passed from the family to residues in the protein of interest. Using MSAs for biological inference is well established,^15^ but as with other methods, is typically repeated manually for each study. Tools exist to visualize genes or proteins with certain types of experimentally or computationally derived annotations such as regulatory sites or structural domains,^16^^,^^17^ as single-gene views of multiple data types,^18–20^ or to target specific diseases such as cancer.^21^^,^^22^ We believe that a more systematic solution can be made for organizing protein-level data across these three categories to assist in the interpretation of human genetic variants.

Existing tools either require the user to translate effects from a DNA or RNA view into an understanding of their potential effect on the encoded protein, do not provide up-to-date information from literature such as derived from NLP, cannot share information across related proteins (eg, PAA), or lack a unified view of diverse annotations. For example, UniProt feature viewer provides some of these layers, but the provenance of alleles, and therefore their disease context, is difficult to ascertain, and the ability to view broad data about an individual protein is separated from the ability to look across isoforms and proteins. The Ensembl variant table has clearer data provenance but lacks NLP resources, and again the per-transcript and pan-transcript views are separate. Therefore, we present P^2^T^2^ as a platform for understanding proteins and protein-coding genetic variation. It is based on an interactive viewer populated using rich genome-wide and proteome-wide functional data including potential phenotypic effects, post-translational modifications, domains, motifs, structure availabilities, literature knowledge derived from NLP-mining, and paralog mappings. We additionally provide mappings to experimentally derived structures, and those of homologs, significantly expanding the ability to identify opportunities to enhance genomic information with 3D structure, which we recently demonstrated is not captured by genomic resources.^23^ We provide P^2^T^2^ as a service, but also a platform which can be customized to each lab or workflow’s needs. P^2^T^2^ is also searchable using Human Genome Variation Society (HGVS) syntax, which is standard nomenclature in clinical genetics reports. Thus, our platform fills an important and currently vacant niche for facilitating the interpretation of human genetic variants and enhancing the information available to research and genetics workflows.

## METHODS

### Transcript mapping

Amino acid sequences of nonfragment isoforms for all human proteins were downloaded from the SwissProt section of the UniProt Knowledgebase,^4^ totaling 46 029 unique mRNA sequences, encoding 33 957 unique isoforms of 19 285 genes. The complete set of transcripts (mRNAs) for these proteins was obtained by matching the protein amino acid sequences from Uniprot to those in the July 2020 release of Ensembl’s Homo Sapiens GRCh38 peptide file (release 100). Transcript identifiers were then used to query Biomart^24^ using the biomaRt R package.^25^ Catalogs of known DNA variants and their annotations built with the bioR software package^26^ were mapped to these DNA sequences using bedtools.^27^ The protein-coding effect of these variants was then annotated using CAVA.^28^

### Annotation data gathering

Protein annotations were integrated from a diverse set of resources including population allele frequencies via gnomAD,^9^ site-specific disease associations through ClinVar^29^ and HGMD,^30^ natural and engineered variants indexed by UniProt,^4^ and post-translational modification sites from PhosphoSitePlus,^10^ and PTMCode2.^11^ Broader features such as domains and motifs were identified using probabilistic models by locally running InterProScan^5^ and ELM.^31^

Hmmer3 alignment^32^ was run for all sequences against the PDB, retaining all pairwise matches or “hits” with a domain significance e-value ≤ 10−5. Hits were sorted by the size of the aligned region and sequence identity within. For simplicity, we chose to display a subset that is locally the best matches available and together provides the most comprehensive coverage of the protein. In order to generate MSAs for use in PAA, human paralogs of each protein were gathered from the Ensembl database. Isoform MSAs were generated for isoforms of each human gene listed in UniProt.

### Software implementation

Annotation data was compiled using custom code from the R programming language (version 3.2.0)^33^ leveraging the IRanges (version 2.2.9),^34^ jsonlite (version 0.9.19),^35^ and doParallel (version 1.0.16)^36^ packages. Data are stored using MongoDB, and served through a REST API built using the Flask Python Framework.^37^ Visual presentation is achieved through a custom-built D3.js (d3js.org; BSD open-source license) implementation^38^ and Bootstrap (getbootstrap.com; MIT open-source license).

## RESULTS

**Figure 1.**
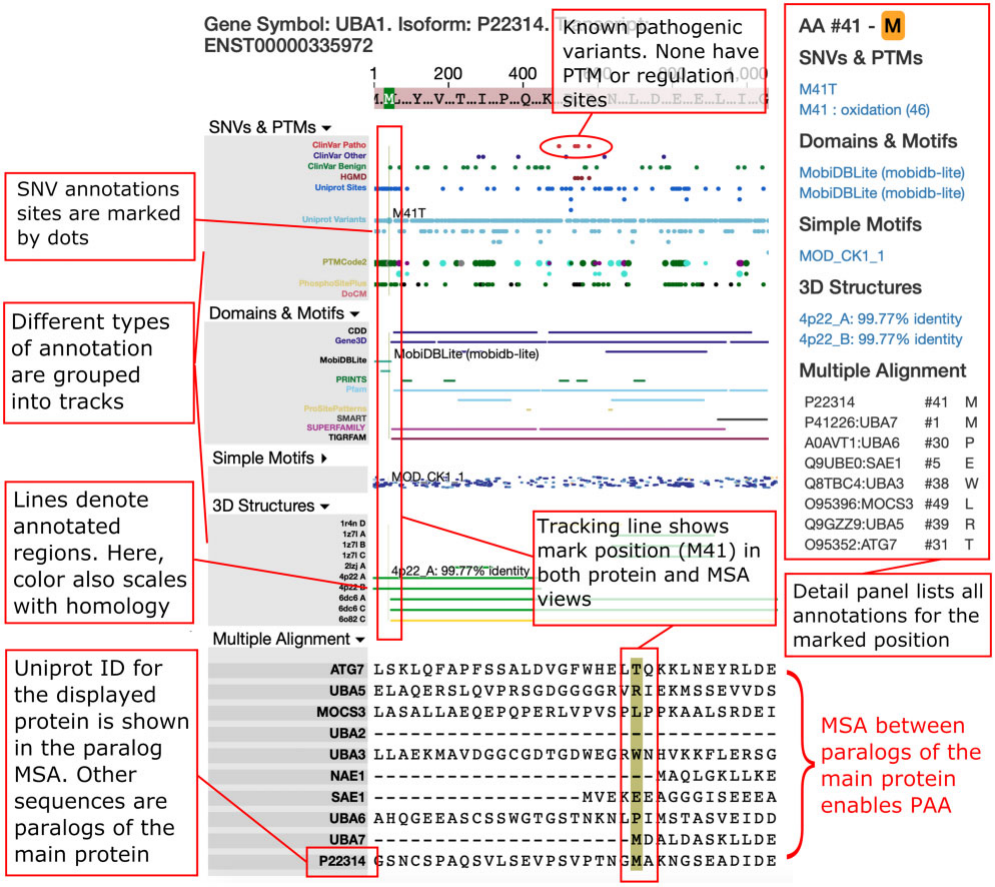
P2T2 for UBA1 demonstrates the rich and comprehensive data that our platform can aggregate and efficiently summarize. When the user places their cursor over an amino acid, the position is highlighted highlight M41 within UBA1, a site with oxidation potential close to the end of an intrinsically disordered region (MobiDB domain is highlighted) and for which homologous experimental structures exist (eg, PDB 4P22 chain A is 99.77% identical). After marking an amino acid, the right-hand panel displays a summary of all available information across that amino acid and the analogous amino acids in the MSA. Color keys for each data type are described in our help page, accessible from the upper toolbar. Pathogenic variants in UBA1 that are associated with muscular dystrophy are noted in the figure. Unlike M41, none of the pathogenic variants are simultaneously annotated with a post-translational regulatory mark.

We present herein our Protein Panoramic annoTation Tool, P^2^T^2^, an interactive web-based tool designed to assist in the interpretation of protein coding variants by presenting multiple annotation and data types in a unified view (Figure 1). Rather than genome-centric, P^2^T^2^ is protein-centric; the data is organized across protein sequences from a wide range of input resources, providing a rich context for evaluating variants at each position of the protein. The tool allows users to search for proteins using many forms of identifier (eg, Ensembl, gene symbol, Uniprot accession, or HGVS mutation nomenclature). Queries that represent nonspecific genomic or protein entities such as gene symbols will be mapped to the protein product encoded by the canonical transcript. Unrecognized queries are mapped to close linguistic matches. Once a protein is selected, P^2^T^2^ provides an interactive interface which can be zoomed by clicking and dragging, and amino acid positions can be “marked” either by entering the position number in a search bar in the header menu, or by shift clicking within the interface. Marking a site creates a mini-report section to the right of the main UI, of all annotations at that position. Thus, P^2^T^2^ facilitates rapid access to protein annotations, for enhancing the interpretation of variants.

Annotation resources used in the default instance of our tool include allele frequencies from gnomAD, variants from ClinVar, and Uniprot, PTMs from PTMCode2, phosphorylation sites from PhosphositePlus, NLP-mined PubMed disease-gene-variant associations from DoCM,^12^^,^^39^ and LitVar,^40^ domains and motifs from ELM and InterProScan (itself a collection of resources), and coverage by experimental 3D structures at varying levels of homology. Tracks that lack data for a given protein are not shown. We provide instructions on how to load additional track data. Each visual element for these data types is linked to its source, providing researchers rapid access to the information most likely to be relevant to the specific variants they are interested in. Finally, the data for each protein includes two multiple sequence alignments: (i) among nonfragment Uniprot isoforms of the selected protein and (ii) among paralogs from Ensembl, making P^2^T^2^ the first automated process for PAA of the human proteome. We believe the combination of annotations available within P^2^T^2^ and the implementation of PAA will help researchers generate novel hypotheses and interpret the effects of missense variants.

### Case example

**Figure 2.**
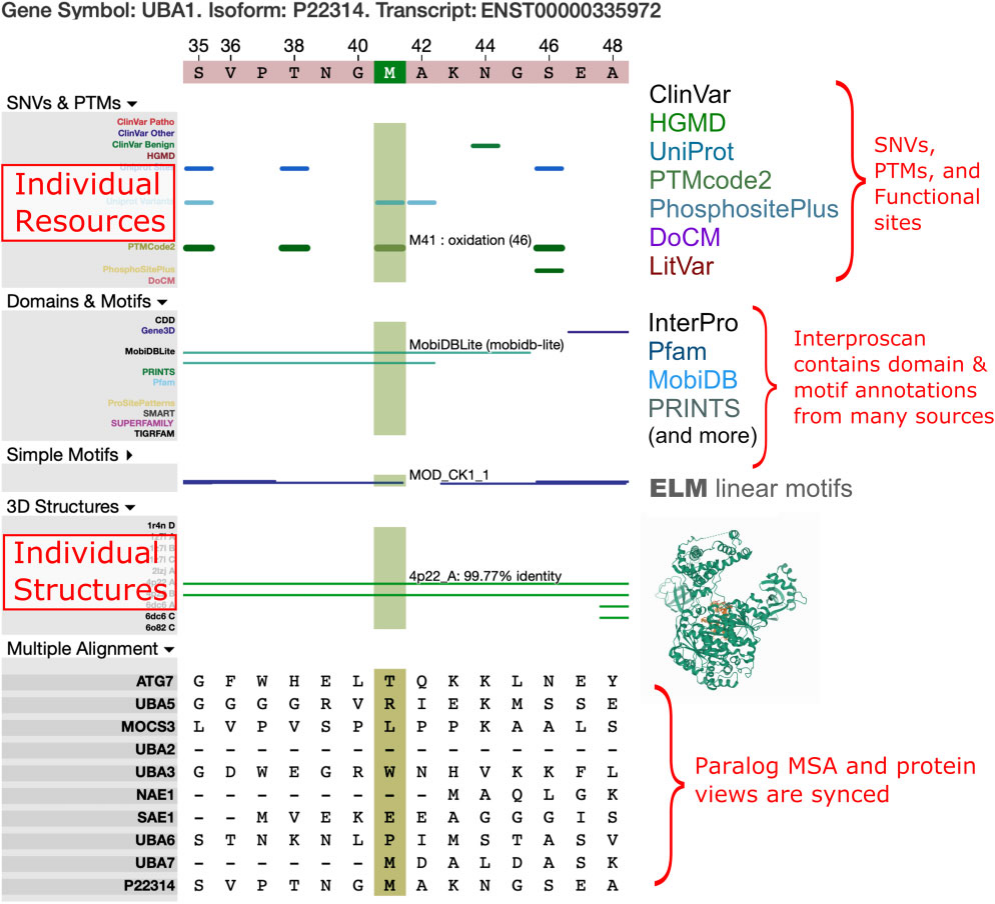
Data are dynamically viewable. Zooming in on the region around M41, the specific and detailed data and annotations available within P2T2 are more easily viewed. We have highlighted M41 and show branding information for many of the available annotations. Domain annotations are provided through Interproscan. Simple Motifs are colored according to their probability of occurrence in random sequences, with blue indicative of higher random probability and orange of lower random probability. For example, the MOD_CK1_1 motif is overlapped by M41. The 3D organization of the protein is important for determining if this motif is accessible; there an experimental structure of UBA1. Clicking on any of the graphical elements directs the user to the corresponding online source data. Finally, the paralog MSA view is synced to the protein view, allowing data from both sources to inform interpretation of positions of interest.

The interface presented with UBA1 selected illustrates how the integrated protein-centric annotations presented by P^2^T^2^ can be useful in hypothesis generation and variant interpretation (Figure 1). Mutation of UBA1 causes an infantile X-linked spinal muscular atrophy. Recently, alternate alleles at M41 have been shown likely to cause an adult-onset autoimmune disorder.^41^ In P^2^T^2^, amino acid position M41 is shown to be a site for post-translational methionine oxidation, which can lead to protein misfolding and regulation^42^—the only such site in the protein. Only UBA1 and UBA7 have a methionine at this position in the MSA among ten human paralog (Figure 2), further supporting a unique function for this amino acid. These features imply that M41 could be a sensitive regulatory site and suggest a mechanism underlying the difference in phenotype for M41 variants from other pathogenic mutations in the same protein. The aggregation of diverse resources in P^2^T^2^ enables connections such as these to be made rapidly, maximizing the utility of variant annotations and facilitating hypothesis generation.

## DISCUSSION

P^2^T^2^ harmonizes diverse information for the interpretation of novel missense variants discovered from next-generation sequencing and clinical genetic testing. The annotations are visually organized to help researchers efficiently identify existing knowledge for specific amino acids, in order to better understand their potential functional effects on the translated exome. Information from primary sequence, domain, motif, and structural levels are presented in a simple format, combining the information available from other tools into PAA across the human proteome. PAA can be used for variant interpretation in the following way: if a novel variant is identified in one protein and the analogous residue in a closely related protein has a similar variant that is known to be pathogenic, this is suggestive of a similar effect in the target protein. The process of leveraging homology to identify sites or regions that are critical for protein functions is well established^13–15^^,^^43^^,^^44^ and has been used for gene function prediction and comparative biology, but only basic recommendations of sequence conservation have been made for VUS interpretation. We access the Database of Curated Mutations (DoCM) and NLP literature resources obtained from LitVar through APIs to ensure the display of up-to-date information; other information is updated on a regular basis using automated loading scripts. P^2^T^2^ combines the annotation of each human protein with annotated MSAs, making this information available systematically and accessible to a wide audience.

In addition to PAA, structural biology is another research process that is not frequently used in the clinical assessment of the potential functional effects of novel variants, or hypothesis generation about their underlying mechanisms. Structural biology and computational biophysics can provide strong indications about the molecular effects of variants.^45–49^ P^2^T^2^ indicates statistically significant relationships to homologous protein structures using sequence profiles, enabling a fuller view of current experimental data and potential for structural modeling. The three-dimensional context provided by these structures can imply a variant’s role in protein function, especially when used in conjunction with the other annotations presented by P^2^T^2^. Thus, we believe our tool will support multiple clinical and research workflows by linking the genomics to 3D experimental and computed structural models, for enhancing interpretation of genetic variation.

Tools have been developed with some features of P^2^T^2^, but our approach has several that are unique and valuable to the field of genetics. Existing tools such as from UniProt aggregate data, but the source and therefore context for most alleles is unclear. Ensembl’s variant table is clear about the source of alleles, but users cannot view the data alongside transcript and paralog tables. Explicit consideration of all transcripts is recommended in the genomic guidelines,^1^ but there are few, if any, tools that support doing so, beyond the linear effects (eg, if missense in one transcript and nonsense in another) available through Alamut^50^ and similar tools. In our tool, the coding effect of each transcript can be viewed, and in the context of the panorama of genomic data. Additionally, we harmonized resources at the DNA level, aiming to avoid discordant interpretations due to different transcripts being annotated. While there have been landmark papers demonstrating significant differences in the interpretation of genetic variants using different transcript resources,^51^^,^^52^ there remains a need to identify which differences among reference protein sequences change the interpretation of human genetic variation. For instance, we feel it is underappreciated that many of the protein-coding transcripts in the databases are not complete but are fragments. We chose to focus on complete isoforms. Of them, 18% have a sequence mismatch between corresponding UniProt and Ensembl sequences. We continue to work on this dimension of the data, planning to leverage ongoing data harmonization efforts by national groups,^53^ and for their implications on genetic variant interpretation. Finally, geneticists and many genomics researchers identify variants in the genome and report them using Human Genome Variation Society (HGVS) nomenclature.^54^ P^2^T^2^ is the first protein-centric tool searchable using genomic HGVS nomenclature. Thus, our tool can be used in automated and manual workflows, with much greater ease and interconnectivity, compared to existing tools. Across the above features and more, P^2^T^2^ provides unique functions that help researchers to interpret the effects of missense genetic variation.

## CONCLUSIONS

P^2^T^2^ is a flexible platform for a truly protein-centric understanding of genomics. It provides a rich environment for understanding each position of a protein by combining annotations of disease-association and functional investigation across all human proteins at amino acid resolution. Our tool can be used to ease the challenge of manual database query and literature review in clinical and research genomics interpretation workflows. Linking the data across gene isoforms and human paralogs enhances how it can be used for interpreting novel genetic variations. Thus, we believe P^2^T^2^ fills a critical role in the expanding repertoire of data science tools for genomics.

## CONTRIBUTORS

M.Z., R.U., and E.K. formulated the concept of the study. E.D., R.Z., and M.Z. contributed software, formal analysis, implementation, and investigation. G.O., P.B., M.C., N.B., and J.K. contributed to data curation and reviewing the written works. J.K., R.U., E.K., and M.Z. supervised aspects of the study. M.Z. completed project administration. J.K., R.U., and E.K. completed funding acquisition.

## Data Availability

Data used by our tool is publically available, unless stated otherwise. The source code and baseline formatted data sets are available at github.com/GenomicInterpretation/p2t2, for users who want to host their own copy of our tool. Instructures for adding new types of data to the tool are provided. We also provide a hosted service, with the link available at the same github page.
